# Harnessing Health Information Technology in Domestic Violence in the United States: A Scoping Review

**DOI:** 10.3389/phrs.2024.1606654

**Published:** 2024-06-21

**Authors:** Vivian Hui, Bohan Zhang, Bomin Jeon, Kwan Ching Arkers Wong, Mary Lou Klem, Young Ji Lee

**Affiliations:** ^1^ School of Nursing, The Hong Kong Polytechnic University, Kowloon, Hong Kong SAR, China; ^2^ Health and Community Systems, School of Nursing, University of Pittsburgh, Pittsburgh, PA, United States; ^3^ School of Nursing, University of Lowa, Iowa City, IA, United States; ^4^ Health Sciences Library System, University of Pittsburgh, Pittsburgh, PA, United States

**Keywords:** health information technology, domestic violence, scoping review, women’s health, domestic abuse

## Abstract

**Objectives:**

The following scoping review aims to identify and map the existing evidence for HIT interventions among women with DV experiences in the United States. And provide guidance for future research, and facilitate clinical and technical applications for healthcare professionals.

**Methods:**

Five databases, PubMed, EBSCOhost CINAHL, Ovid APA PsycINFO, Scopus and Google Scholar, were searched from date of inception to May 2023. Reviewers extracted classification of the intervention, descriptive details, and intervention outcomes, including physical safety, psychological, and technical outcomes, based on representations in the included studies.

**Results:**

A total of 24 studies were included, identifying seven web-based interventions and four types of abuse. A total of five studies reported safety outcomes related to physical health. Three studies reported depression, anxiety, and post-traumatic stress disorder as psychological health outcomes. The effectiveness of technology interventions was assessed in eight studies.

**Conclusion:**

Domestic violence is a major public health issue, and research has demonstrated the tremendous potential of health information technology, the use of which can support individuals, families, and communities of domestic violence survivors.

## Introduction

Domestic violence (DV) is a significant yet frequently underreported public health concern that menaces individuals’ physical and mental wellbeing globally [1]. As defined by the United States (U.S.) Office on Violence against Women, DV encompasses a pattern of abusive behavior within any intimate relationship that one partner uses to sustain or maintain control over another partner [2]. An array of abuse that could happen within a household includes physical, psychological, sexual, and financial towards the children, elderly, or intimate partner [2]. One in three women encounters physical violence from an intimate partner every minute, resulting in approximately 10 million abuse survivors annually in the U.S. [3]. Moreover, DV exerts a significant financial burden with an estimated cost of $103,767 per female affected by intimate partner violence (IPV) [4].

Compared to men, women exhibit a significantly elevated vulnerability to experiencing DV in a lifetime [5]. Persistent experience of DV is associated with an array of detrimental effects on physical and mental health, including hypertension, diabetes [6], depression, anxiety, and post-traumatic stress disorder (PTSD) [7–9], AIDS/HIV infection [10], sleep disturbances [11], and suicide attempts [12]. Nevertheless, the profound impact of DV on women is often underestimated due to its intricate nature, the prevalence of revictimization, and intergenerational victimization [13].

Intervention studies are pivotal in empowering survivors to overcome their challenges and enhancing their physical and mental wellbeing. However, conducting studies involving women with DV experiences often encountered substantial challenges. Previous research highlighted the difficulties faced when approaching DV survivors in person due to societal stigma, shame, guilt, alienation, and judgment [14–16]. Traditional intervention methods such as focus group, one-to-one in-person interviews, and individual/group therapy require substantial time for participants’ recruitment and are susceptible to confidentiality issues among participants. Recently, health information technology (HIT) has been gaining traction in DV research, such as developing more precise and effective screening tools and improving the overall wellbeing for DV survivors. HIT refers to the utilization and application of information processing that includes both computer hardware and software, across the spectrum of data storing, retrieving, sharing, and using healthcare information, health-related data from both patients or healthcare providers, and knowledge for effective healthcare communication and decision-making in certain disease journey [17]. HIT interventions (e.g., guided online support) offer an anonymous environment that allows survivors to access information and seek help without the time and location constraints [18]. Women with DV experiences face additional vulnerabilities due to limited access to resources and the societal-asserted feminine duties on childcare and housework tasks. The use of HIT intervention allows these women to reach resources and seek help from the virtual space without restrictions. Also, studies indicated that women survivors tend to be more emotional and prefer text-based communication with healthcare providers as it provides them with a sense of anonymity and security [19].

With HIT accessible through mobile devices, women may easily acquire a wealth of health information and increase their likelihood of disclosing their abusive experience [20]. Studies also show the use of HIT interventions can improve health outcomes among the DV population. However, to the best of our knowledge, current reviews have only examined HIT interventions for mental health [21], child maltreatment [22], and peer aggression [23]. Although the application of HIT in the DV population has surged unprecedentedly, no evidence summarized the current state of the science of HIT interventions particularly in women with DV experiences.

As such, this scoping review aims to identify and map the available evidence for HIT interventions among women with DV experiences in the United States. This review can guide future research and advance the clinical and technological applications available to healthcare professionals.

## Methods

### Search Strategy

Four bibliographic databases were searched from date of inception to May 2023 (PubMed, EBSCOhost CINAHL, Ovid APA PsycINFO, Scopus). Initial searches were run in Oct 2018 and search updates were run in May 2023. Searches were also run in Google Scholar. To ensure comprehensive coverage, a health sciences librarian (blinded) designed the PubMed search strategy and then adapted that search strategy for use in other databases. The search strings utilized a combination of natural language and, where applicable, controlled vocabulary to cover the concepts of “technology” and “family or domestic violence.” In instances where possible, the search results were limited to articles published in the English language. The full list of search strings from different databases is shown in Table 1.

**TABLE 1:** Search string from different search engines (Worldwide publications, from date of inception to May 2023).

Search engine	Search string
PubMed	(("Mobile Applications"[Mesh] OR "Internet"[Mesh] OR "Cell Phone"[Mesh] OR "Videoconferencing"[Mesh] OR "Crowdsourcing"[Mesh] OR smartphone[tiab] OR app[tiab]) AND ("Intimate Partner Violence"[Mesh] OR "Gender-Based Violence"[Mesh] OR "Domestic Violence"[Mesh] OR intimate partner violence[tiab] OR domestic violence[tiab] OR child abuse[tiab] OR spouse abuse[tiab] OR elder abuse [tiab]))
PsycINFO	((online social networks/OR social media/OR online community/OR mobile application*.ti,ab. OR exp mobile devices/OR text messaging/OR computer applications/OR exp electronic communication/OR (smartphone* or app).ti,ab.) AND (domestic violence/or child abuse/or elder abuse/or emotional abuse/or intimate partner violence/or marital conflict/or partner abuse/OR (domestic abuse or domestic violence or intimate partner violence or child abuse or spouse abuse or elder abuse).ti,ab.)) NOT (0200.pt. OR 0240.pt. OR 0280.pt. OR 0400.pt.)
CINAHL	((TI mobile application* OR AB mobile application* OR TI app OR AB app OR TI smartphone* OR AB smartphone* OR TI facebook OR AB facebook OR TI Twitter OR AB Twitter OR TI social media OR AB social media OR TI social network* OR AB social network* OR MH "Mobile Applications" OR MH "Cellular Phone" OR MH "Text Messaging" OR MH "Smartphone+" OR MH "Crowdsourcing" OR MH "Online Services") AND (TI domestic violence OR AB domestic violence OR TI domestic abuse OR AB domestic abuse OR TI intimate partner violence OR AB intimate partner violence OR TI spouse abuse OR AB spouse abuse OR TI child abuse OR AB child abuse OR AB elder abuse OR MH "Domestic Violence+")) NOT PT dissertation
Scopus	("domestic violence" OR "domestic abuse" OR "intimate partner violence" OR "spouse abuse" OR "child abuse" OR "partner abuse" OR "gender based violence" OR “elder abuse”) ("mobile application*" OR app OR smartphone* OR "cell*phone*" OR "text messag*" OR "smartphone*" OR "crowdsourcing")
Google Scholar	"Mobile Applications" OR "Internet"OR "Cell Phone" OR "Videoconferencing" OR "Crowdsourcing" OR ''smartphone'' OR ''app'' AND "Intimate Partner Violence" OR "Gender-Based Violence" OR “Elder abuse”

### Definition of DV

In this article, abuse that could happen in a household setting in the United States, including child abuse, IPV and elder abuse, regardless of the relationship between survivors and perpetrators will be considered as DV. Specifically, DV can occur between a parent and child, or siblings, while IPV can only occur between romantic partners who may or may not be living together in the same household.

### Eligibility Criteria

The inclusion criteria include 1) health information technology interventions applied 2) women with DV experiences (including IPV, child abuse and elder abuse) 3) published between January 2008 and May 2023. Studies related to dating violence and social media were excluded because the settings could be out of the household in the United States. Editorials, commentaries, non-peer reviewed articles, study protocols, conference abstracts, and non-English articles were also excluded.

### Review Process

**FIGURE 1:**
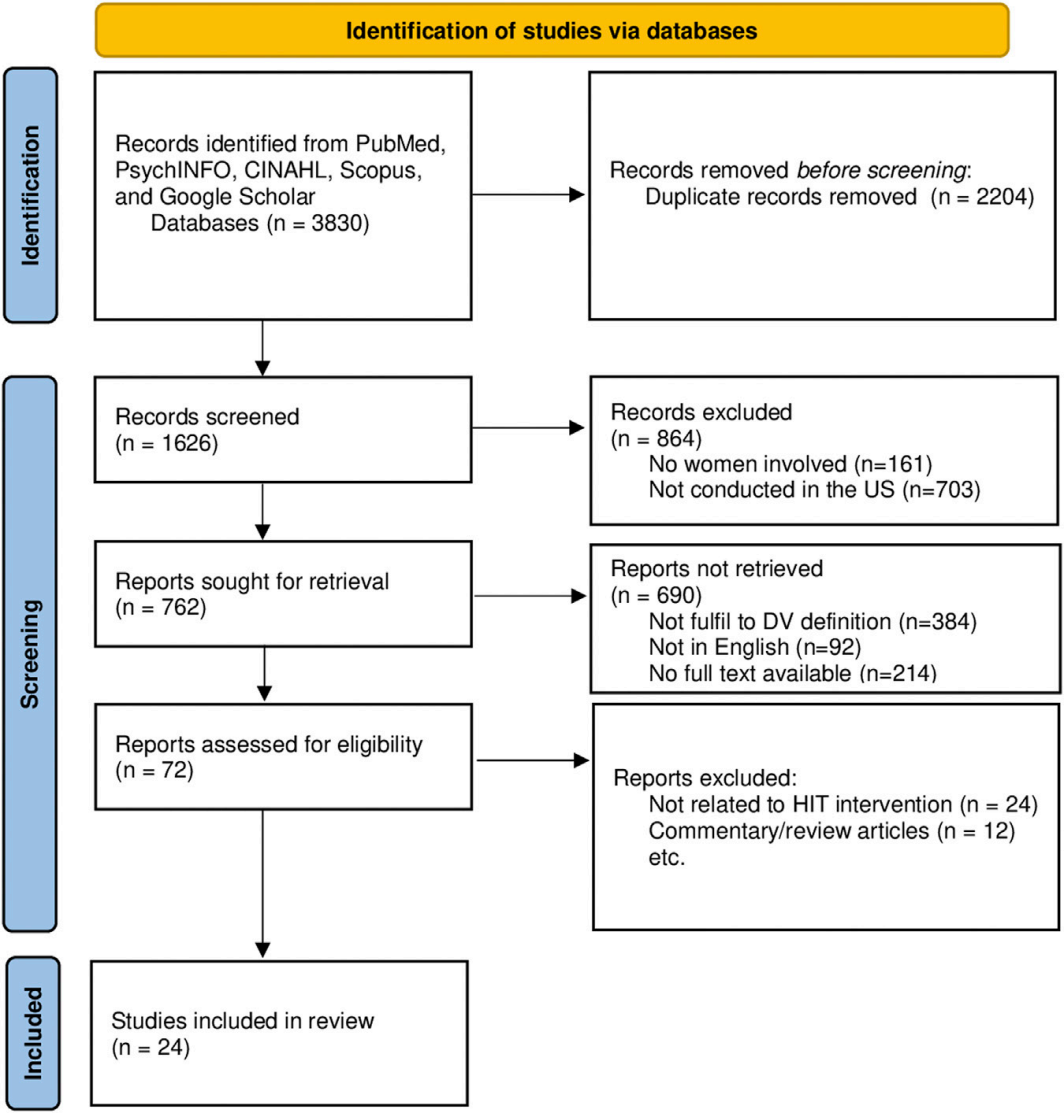
Preferred reporting Items for Systematic Reviews and Meta-Analyses (PRISMA) flow diagram (Worldwide publications, from date of inception to May 2023).

In the initial phase (Figure 1), articles were assessed based on their titles and abstracts, specifically examining their relevance to technology interventions for DV. Articles did not include women, or not conducted in the United States were not retrieved. Subsequently, in the second stage, full-text documents were obtained and subjected to qualitative review, guided by inclusion criteria. Prior to screening, a training session was conducted where reviewers evaluated a set of sample records together. A 75% inter-rater agreement was achieved among reviewers on this initial set before proceeding to the formal screening. During the formal screening, two independent reviewers examined the titles and abstracts of the records and categorized them as “included,” “excluded” or “uncertain” based on the relevancy to HIT interventions for DV. The reasons for exclusions include not related to HIT intervention, review articles, or any commentary articles. For any uncertain records, a senior researcher (BLINDED) resolved the status of the record.

### Data Abstraction

Two reviewers independently charted data from each eligible articles. Any disagreements were resolved through group discussions or adjudication by a third author. The variables in the spreadsheet were organized into three categories: 1) classification of the intervention (including study purpose, technology, type of intervention, and abuse), 2) descriptive details (such as sample size, location, and settings), and 3) intervention outcomes (including physical safety, psychological, and technological outcomes). This scoping review followed the guidelines from Arksey and O’Malley [24].

## Results

A total of 3,830 articles were identified from four academic databases, as well as from Google Scholar. 2,204 duplicates and irrelevant articles were removed, and 72 articles underwent a more detailed screening based on the abstract and titles. 48 articles were subsequently removed due to irrelevance to the research questions or ineligibility to the predefined criteria (e.g., non-intervention studies, exploratory studies conducted in non-U.S. territory). Finally, twenty-four articles (N = 24) met all of the inclusion criteria and were included in this review (Figure 1). Table 2 listed the participant characteristics, purpose of study, purpose of intervention and types of technology, while Table 3 described the outcomes of interventions measured across studies.

**TABLE 2:** Literature summary of critical findings among 24 studies from 2008 to 2023 (Worldwide publications, from date of inception to May 2023).

No.	Authors (Year)	Purposes of the study	Types of technology	Purpose of intervention	Participants characteristics			
					Sample size	Location	Races	Age (mean)
1	Bacchus, et al (2016)	Explore perinatal home visitor’s and women’s perceptions and experiences of the Domestic Violence Enhanced Home Visitation Program (DOVE) using mHealth technology or paper-based method.	Domestic Violence Enhanced Home Visitation Program (DOVE) mHealth Technology	Exploration	N = 26	Metropolitan area	White, Black, Mixed	20–27 mainly
2	Bloom, et al (2014)	Evaluate the feasibility of Internet-based safety planning for rural and urban abused pregnant women and practicality of recruitment procedures for future trials.	Online safety planning intervention	Prevention	N = 46	N/A	White	25
3	Blumling, et al (2018)	Evaluate a standardized patient simulation experience depicting a victim of IPV on undergraduate nursing student knowledge and confidence in assessment and intervention of IPV	Standardized patient simulation	Education	N = 57	N/A	N/A	N/A
4	Choo, et al (2016)	Examine the feasibility and acceptability of a computer-based program and telephone booster for drug-using women reporting IPV.	Web-based BSAFER intervention and booster phone calls	Education	N = 40	Metropolitan area	Non-white, Hispanic/Latino	25
5	Constantino, et al (2015)	Compare the effectiveness of online, face-to-face and waitlist control intervention of the HELPP based on personal, interpersonal and community level	Online, face-to-face, Wait-list control HELPP intervention “Email”	Education	N = 32	Metropolitan area	White, Black, Asian	40
6	Eden, et al (2015)	To test the effectiveness of a safety decision aid compared with usual safety planning (control) delivered through a secure website, using a multistate RCT design.	Internet safety decision aid	Prevention	N = 708	Metropolitan area	White Black Asian Native American Hawaiian or Pacific Islander Other Multi-racial	33
7	Ejaz, et al (2017)	Comparing the managers’ knowledge change after receiving educational online training modules about the background of abuse, screening and reporting abuse.	Online training modules	Education	N = 453	Metropolitan area	N/A	N/A
8	Glass, et al (2017)	To compare safety and mental health outcomes at baseline, 6 months, and 12 months among abused women randomized to (1) a tailored, internet-based safety decision aid or (2) control website.	Internet safety decision aid	Prevention	N = 672	Metropolitan area	White Black Asian Native American Hawaiian or Pacific Islander Other Multi-racial	33
9	Goldman, et al (2019)	Examine the knowledge level and feasibility of using a smartphone application to identify victims of sexual exploitation.	SART START smartphone application	Assessment	N = 103	N/A	White Asian Black Other	31–40 mainly
10	Gur, et al (2016)	Explore the use of GPS for domestic violence or Intimate Partner Violence in pretrial programs	GPS	Exploration	N = 114	Metropolitan and rural areas	White	70% were 40 or older
11.	Harris, et al (2009)	Evaluate the costs and effectiveness of promoting online CME about IPV training to physicians.	Free CME online program	Education	N = 1869	Metropolitan area	N/A	N/A
12	Hassija, et al (2010)	To evaluate the effectiveness and feasibility of videoconferencing technology to provide evidence-based treatment to rural domestic violence and sexual assault populations	Videoconferencing	Prevention	N = 13	Metropolitan and rural areas	White	30
13	Ibarra, et al (2014)	To examine “styles of surveillance” among community corrections officers using Electronic monitoring, by employing a specific and comparative analysis from GPS in DV in the context of pretrial supervision	GPS	Exploration	N = 50	Metropolitan and rural areas	N/A	N/A
14	Jabaley, et al (2011)	Examine the iPhone™ when used as an assessment tool and an enhancement to an evidence-based, in-home child safety intervention.	iPhone™	Assessment	N = 3 families	Metropolitan area	N/A	N/A
15	Lefever, et al (2008)	Assess the feasibility of using cell phone interviews to learn more about the quality of daily parenting and child neglect.	Cell phone interview	Screening	Study 1:N = 45 Study 2: N = 544	Metropolitan area	African European Hispanic, other ethnic	Adolescent mother: 17.5 Adult mother: 26.5
16	MacLeod, et al (2009)	To assess whether the telemedicine would increase the ability of the rural provider to perform a complete and accurate sexual assault examination.	Telemedicine video-conferencing	Assessment	N = 42	Rural area	N/A	7
17	McAndrew, et al (2014)	To determine whether the dentistry’s online tutorial on domestic violence is effective for dental students poised to embark on their professional careers	Online tutorial	Education Prevention Detection	N = 25	Metropolitan area	N/A	N/A
18	Paranal, et al (2012)	To discuss the benefits and limitations of conducting online organizational trainings from the perspective of participants, including what participants found effective, what challenges were most commonly encountered, and trainee perspectives of the program’s overall impact	Online training	Education	N = 218	Metropolitan area	N/A	N/A
19	Rothman, et al (2009)	To assess the proportion of battered women’s shelter residents who use e-mail in communication	E-mail	Assessment	N = 57	Metropolitan area	White Black Hispanic Asian, others	30
20	Sargent, et al (2016)	To assess the effects of an online program (Change A Life) designed to educate individuals about children’s exposure to domestic violence, and to increase individuals’ self-efficacy for providing support to children exposed to DV.	Online program	Education Prevention	N = 255	Metropolitan and rural area	White, Black, Hispanic Asian	39
21	Thraen, et al (2008)	Evaluate the usability and satisfaction differences on a Web-based application developed for the remote sharing of child maltreatment assessment.	Web-based application TeleCAM	Assessment	N = 11	Metropolitan area	White African American	N/A
22	Abujarad, et al 2021	Developed and evaluated the usability of a self-administered digital health tool, VOICES, that can be used to screen, educate, and motivate older adults to self-report elder abuse.	A tablet-based self-administrated digital health tool	Education Assessment Self-identification Interview	N = 38	Metropolitan area	African, American Asian White Other	61–98
23	Agu, et al 2020	Improve intimate partner violence service delivery.	Two days of interactive sessions, six webinars, testing strategies using the improvement model (Plan-Do-Study-Act) and three online surveys	Education Assessment	N = 98 (IPV home visitors)	N/A	N/A	N/A
24	Bagwell-Gray, et al 2021	Adapt and test the myPlan web app intervention for Native American women.	Smartphone application	Exploration	N = 83	Metropolitan and rural areas	N/A	N/A

**TABLE 3:** Literature summary of critical findings among 24 studies from 2008 to 2023 (Worldwide publications, from date of inception to May 2023).

No.	Authors (Year)	Types of abuse	Study design	Intervention outcome		
				Physical safety outcomes	Psychological outcomes	Technology outcomes
1	Bacchus, et al (2016)	IPV	Qualitative Interviews	N/A	N/A	“The computer tablet viewed as a safe and confidential way for DV women to disclose their experiences; tablet helped to establish trust and rapport between victim and providers; The technology helped reduce the anticipated stigma associated with disclosing abuse.” “Both methods of screening were positively influenced by factors like having established trust and rapport, and good interpersonal communication.”
2	Bloom, et al (2014)	IPV	Feasibility	Danger assessment score indicated severe danger in the abusive relationship. (M = 16.1)	N/A	The average time to completion was 10.3 days (SD = 16.3 days, range = 0–68 days), with rural women taking an average of 2.2 days longer than urban women (11.6 vs. 9.4 days, respectively). A higher percentage of rural women (63.2%) reported using a home computer compared with their urban counterparts (48.4%). A lower percentage of rural women used a computer at a friend’s or family member’s house (25.3% vs. 34.3, respectively) 20% of e-mail contacts from potential participants originated from a mobile device. 19.5% of the women completed the baseline session from a mobile device.
3	Blumling, et al (2018)	IPV	Cross-sectional study	N/A	N/A	The simulation technology demonstrated significant increase in confidence (*p* < 0.001), and knowledge (*p* < 0.001).
4	Choo, et al (2016)	IPV	RCT	Past month drug use, the occurrence of psychological, physical, and sexual violence.	N/A	The web-based intervention plus telephone is highly feasible in the emergency care setting; High acceptability, satisfaction, and usability in the web-based intervention evaluation Mean System Usability Scale (SUS) for the BSAFER Web program was 84 (95% CI 78–89) of 100; mean Client Satisfaction Questionnaire (CSQ-8) was 28 (95% CI 26–29) of 32.
5	Constantino, et al (2015)	IPV	Sequential, transformative mixed-methods design	N/A	Significant improvement in anxiety (*p* = 0.01), depression (*p* < 0.001) and social support (*p* < 0.001) in the pre-test score and post-test score for each of the groups; Online intervention may lessen social risks and inhibitions, enhances sharing of unwelcome thoughts and painful feelings.	
6	Eden, et al (2015)	IPV	RCT	Intervention-group women had a greater reduction in total decisional conflict, uncertainty and feeling unsupported than control participants (*p* < 0.05).	N/A	N/A
7	Ejaz, et al (2017)	Elder abuse	Feasibility study	N/A	N/A	Significant improvement in knowledge for abuse background and report system (67%); The module “Screening of abuse” is the weakest module as it lacks illustrations relating to various types of self-neglect (environmental, health-related, and behavioral)
8	Glass, et al (2017)	IPV	RCT community-based	Women who left the abuser had higher baseline risk (*p =*0.003); they found the safety behaviors more helpful (*p* = 0.037) and experienced less decisional conflict (*p* = 0.042).	Women in intervention group had greater reductions in psychological IPV (*p* = 0.001) and sexual IPV (*p* = 0.001).	N/A
9	Goldman, et al (2019)	Child abuse	Feasibility	N/A	N/A	Knowledge increased significantly in smartphone application intervention group; the application reported as easy to use (59%), useful (63.9%) and preferred to obtain information than traditional printed materials (85%).
10	Gur, et al (2016)	IPV DV	Feasibility	N/A	N/A	The GPS in DV is important in providing enhanced supervision (96%), keeping victim safer (94%), allowing defendants to live in the community while awaiting trial (86%), receiving satisfaction monitoring information and supervising their clients easier for officers (51%); GPS technology facilitated asking’ hard questions and help guiding client’s decision making (92%).
11.	Harris, et al (2009)	IPV	N/A	N/A	N/A	The overall quality of the online CME program was rated highly in knowledge improvement (M = 4.52/5); Direct email was the most effective, cost efficient, and common strategy of online intervention recruitment.
12	Hassija, et al (2010)	DV	N/A	N/A	Participants exhibited large reductions on PTSD severity checklist (M = 32.20, SD = 12.68, *d* = 1.17). And depressive symptoms (M = 13.07, SD = 9.07, *d* = 1.24); Clients’ reports of satisfaction with the provision of psychological services via videoconferencing (M = 52.93, SD = 2.43).	Videoconferencing is effective in providing specialized, evidence-based psychological services to rural domestic violence and sexual assault populations.
13	Ibarra, et al (2014)	DV	Qualitative interview/comparative analysis	N/A	N/A	The GPS is useful in tracking location, building trustful relationship, facilitating the interviews, and being a source of solace against the threat of false accusation.
14	Jabaley, et al (2011)	Child abuse	N/A	Observation System: The Home Accident Prevention Inventory-Revised (HAPI-R) demonstrated significant decrease in hazards among families (Range: 74%–97%); Parents considered their homes safer and expressed confidence in recognizing and securing hazards.	N/A	Communication via iPhone on logistical questions or reminders demonstrated good results; 86% Reported with favorable reactions to the iPhone texting communication.
15	Lefever, et al (2008)	Child Abuse	Longitudinal study	Higher parenting essentials associated with higher knowledge of child development, higher scores on the parenting styles measure, lower child abuse potential, and lower scores on the history of neglect measure.	N/A	Cell phone offers greater level of mobility and convenience at a lower cost than landline phones; reported as useful in intervening with mothers at risk of suboptimal parenting and child neglect.
16	MacLeod, et al (2009)	Child abuse	N/A	N/A	Rankings of practitioners’ skills and the telemedicine consultation effectiveness were high, with 82% of cases.	Telemedicine consultations showed good scores in the use of the multimethod examination technique and the use of adjunct techniques. The mean duration of the consultations was 71 min (range: 25–210 min). The consultations resulted in changes in interview methods (47%), the use of the multimethod examination technique (86%), and the use of adjunct techniques (40%). There were 9 acute sexual assault telemedicine consults that resulted in changes to the collection of forensic evidence (89%). Rankings of practitioners’ skills and the telemedicine consult effectiveness were high, with 82% of cases scoring ≥5 on a 7-point Likert scale.
17	McAndrew, et al (2014)	IPV	Quasi-experimental	N/A	N/A	“The online tutorial was effective in increasing the participants’ perceived preparation, knowledge, and self-efficacy and decreasing perceptions of provider constraints in managing victims of IPV.”
18	Paranal, et al (2012)	Child abuse	Non-equivalent group design	N/A	N/A	Individual participants rated the training content (9.13) and found the training format interesting (8.88), useful (8.9) and ease of use above neutral (6.4). 80% of the participants viewed all the video clips. Organization perceived online training as easy to administer to staff (Mean:7.6), prefer online training (Mean:7.25); Effective in teaching adults about child abuse (Mean:8.97).
19	Rothman, et al (2009)	IPV	N/A	N/A	N/A	Most shelter residents have email accounts (80%), prefer using email as communication and keep in touch with the advocates; 88% reported as a safe method.
20	Sargent, et al (2016)	DV	RCT	N/A	Knowledge about DV consequences, self-efficacy, and how to help children exposed to DV reported as significant improvement in intervention group.	Online program is effective to reach large numbers of people inexpensively and quickly raise public awareness of DV effectively, offer a cost-effective way and allow participants to move through the program at their own pace.
21	Thraen, et al (2008)	Child abuse	Mixed methods	N/A	N/A	85% Participants used desktop PCs on a regular basis; reported the easiness to save and upload images from Web browser (81%); able to download and install plug-ins (72%); E-mail used for assessing child maltreatment (55%).
22	Abujarad, et al 2021	Elder abuse	Mixed methods	N/A	N/A	Overall scored the VOICES usability score of 86.6, while 93% participants indicated the willingness to recommend digital VOICES tool to others and 100% indicated full understanding of health information and content.
23	Agu, et al 2020	IPV	Mixed methods	N/A	N/A	From baseline to final survey, participants reported accurate knowledge (change: 2.3%–34.8%), confidence (change: 31.8%–37.9%), system awareness (change: 22.7%–53.5%), and increased IPV screening rate (change: 88.0%–91.0%) and referrals (change: 43.0%–100.0%).
24	Bagwell-Gray, et al 2021	IPV	Qualitative interview	N/A	N/A	By understanding IPV’s culture specific risks and protective factors, the web-based safety app called myPlan (renamed ourCircle) can better provide IPV Native American women with culturally specific safety priorities and security strategies.

Note: DV, domestic violence; IPV, intimate partner violence; M, mean; RCT, randomized controlled trial; SD, standard deviation; PTSD, Post-traumatic stress disorder.

### Types of Technology

Seven web-based interventions were identified; five of them collected data from a stand-alone website [25–29]. Four online trainings were designed for multidisciplinary healthcare professionals [30–33]. Five studies leveraged mobile devices to provide intervention [34–38]. Two studies used email as an intervention tool [31, 39]. Another two studies used Global Positioning System (GPS) as interventions [40, 41]. One article used videoconferencing as intervention [42], and another study utilized simulation technology to tackle domestic violence [30].

### Types of Intervention

Four studies focused on prevention [25–27, 42], and ten focused on education [28, 30–33, 37, 39, 43–45]. Five studies delineated the effectiveness of HIT intervention [34, 40, 41, 46, 47] and three focused on various types of DV assessment [29, 35, 48]. Only one study emphasized screening for patients [36].

### Participant Characteristics

Different types of DV survivors or potential survivors were captured in this review, such as adolescent mothers [36], abused women [27, 42], pregnant women [25, 46], and battered women [47]. Simultaneously, participants were extended to various professions like nurses [30], physicians [31], dental students [32], and law enforcement officers [34]. Among twenty-four studies, the sample sizes were ranged from 11 to 1,869. The sample characteristics and demographics were diversified. The majority of studies recruited participants from designated clinical settings (e.g., hospitals, clinics), community centers (e.g., non-governmental organizations, shelters), schools, and criminal justice services departments. A mean age of 7–40 was found across studies.

The review studies include people of diverse ethnic backgrounds, including Whites, African Americans, Hispanic Latinos, Asians, and others; however, the samples were predominantly White (N = 13). Only one article was conducted in rural areas [48] compared to fourteen articles in metropolitan areas [26, 27, 29, 31, 32, 35–37, 39, 43–47]. Another five articles have been discussed domestic violence in both metropolitan and rural areas [28, 38, 40–42].

### Types of Abuse

Four types of abuse were included. Twelve studies focused on intimate partner violence (IPV) [25–27, 30–33, 38, 39, 43, 46, 47], six highlighted child abuse [29, 34–36, 45, 48], four illustrated DV [28, 40–42], and two studies targeted on elder abuse [37, 44].

### Study Designs

Various research methods are used, including randomized controlled trials (N = 5) [26–28, 43, 46], and longitudinal (N = 1) [36], mixed methods (N = 5) [29, 33, 37–39], feasibility tests (N = 5) [25, 30, 34, 40, 44], non-equivalent group design (N = 1) [45], quasi-experimental design (N = 1) [32], as well as qualitative studies (N = 1) [41].

### Intervention Outcomes

#### Physical Safety Outcomes

Physical safety outcomes were reported in five studies [25–27, 36, 43]. Only one study assessed the abuse score for physical sexual violence [43], while the other one evaluated the neglect score among children at a home setting [36]. Both studies indicated a decrease in scores for both neglect and abuse. Moreover, three other studies showed improvement in danger assessment, safety strategies, and safety behaviors using different types of measurements [25–27].

#### Psychological Outcomes

Depression, anxiety, and PTSD were reported as psychological health outcomes in three articles [25, 39, 42]. Bloom et al. (2014), Constantino et al. (2015), and Hassija et al. (2010) showed improvement in depression [25, 39, 42], Constantino et al. (2015) and Bloom et al. (2014) also reported improvement in PTSD and anxiety outcomes in their studies [25, 39]. Choo et al. (2016) and Lefever et al. (2008) illustrated the abuse reduction as an outcome measure [36, 43]. Only one article reported improved social support [39].

#### Technological Outcomes

Technological interventions effectiveness was evaluated in eight studies, which demonstrated strong feasibility, usability, and acceptability [25, 29, 34, 36, 37, 42–44]. Bloom et al. (2004), Choo et al. (2016), Thraen et al. (2008), and Abujarad et al. (2021) measured the usability [25, 29, 37, 43], while Bloom et al. (2014), Choo et al. (2016), Ejaz et al. (2017), Goldman et al. (2019), Hassija et al. (2010), and Lefever et al. (2008) measured feasibility [25, 34, 36, 42–44], and Choo et al. (2016) measured the acceptability of the technological intervention [43].

## Discussion

Our study synthesized research articles on the technology interventions, research designs, and reports on the overall state of DV technology over the past decade. The results demonstrated the diverse application of technological interventions among women with DV experiences. Seven types of technology interventions were identified for DV (e.g., mobile applications, online training, web-based intervention, Global Positioning System (GPS), emails, videoconferencing, and simulation), and the majority of studies assessed usability, acceptability, and participants’ satisfaction. Several studies assessed psychological wellbeing outcomes, including stress levels, quality of life, sleep quality, and emotional needs.

### HIT Interventions Dominant in IPV

Our results highlight that HIT interventions focused on different types of abuse within families, with Intimate Partner Violence (IPV) being the most common and elder abuse being the least. Research has demonstrated that young and educated people were the population group more willing to accept and adopt HIT interventions in clinical and community settings, while older adults staying at home were unwilling to use these health interventions due to perceived barriers, such as inertia, cognitive impairment, and physical disabilities [49, 50]. However, this situation might decline because of aging and the rise of computer literacy among the elderly. According to Internet World Statistics, over 89 percent of the U.S. population were internet users in 2019 [51]. The web-literate population has been growing and constantly surging with the increasing reliance on technology, machine learning, and artificial intelligence in our daily lives. Fleming (2015) delineated that over 60% of people older than 50 used technologies to browse social networking sites, take photos, and communicate in text messages [52]. Although those older than 65 may have more perceived barriers to using health technology, such as hearing deficit and cognitive impairment, they will accept new technologies when the benefits outweigh the drawbacks. There has been a proliferation of research concerning the utilization of technology in addressing elder abuse, highlighting its significant feasibility, accessibility, and effectiveness in monitoring neglect or physiological changes among elder abuse survivors, both at home and in clinical settings [53]. Therefore, it is anticipated that computer literacy and the implementation of HIT interventions among the elderly will continue to grow, leading to the expansion of interventions targeting aging in the forthcoming decades.

### HIT Interventions Thrives in Urban and Rural Areas

The findings of our study also revealed that the use of HIT had been employed to assist DV survivors residing in both urban and rural settings. Due to limited access to healthcare services, women residing in rural areas experiencing DV face difficulties in effectively addressing their DV situation [54]. Previously, individuals from rural areas who were DV survivors may not have benefitted from technological advancements and treatments due to the inadequate support available to them [54]. However, our review demonstrates that digital devices in rural areas hold promise for telemedicine, providing an interactive means of delivering care, making referrals, and offering diagnosis and screening for DV survivors who may be able to meet healthcare professionals physically in a clinic or hospital. As the digital literacy rate has consistently improved over the past decade, it is expected that the limitations of technical support (such as limited wireless devices, unstable Internet, and restricted access to technical assistance) are no longer hindrances to implementing HIT interventions in rural areas. The current body of research has explored the utilization of videoconferencing, interventions pertaining to medical appointments, counseling services, and danger assessments, all of which should be expanded to rural areas through digital platforms or means. It is anticipated that the utilization of teleconferencing and online counseling services will become even more prominent, as indicated by the expanding coverage of health insurance plans for online services and consultations that aimed at addressing the mental health needs of women with DV experiences.

### Web-Based Interventions Provide Better Platform for Self-Disclosure

The utilization of web-based interventions has emerged as a growing trend in DV prevention and education. HIT offers several advantages, including convenience, interactive design, and the creation of an anonymous environment facilitated by the Internet. Given that DV survivors may be reluctant to engage in face-to-face social interactions due to fear of stigmatization, shame, guilt, and judgment [16], web-based interventions provide an anonymous platform that encourages survivors to disclose their traumatic experiences more vividly and seek help proactively. It is well-documented that the sense of anonymity and social distance feeling created by the online platform fosters candid, sensitive and traumatic conversations [55, 56]. It is noteworthy that effective patient-provider communication remains the linchpin of positive intervention outcomes, particularly when addressing sensitive topics such as DV. Technological advancements promise to promote truthful or effective communication between DV survivors and healthcare professionals.

While utilizing technology for DV was predominantly applied at the patient level, the application also extends to healthcare professionals’ training. Previous literature highlighted the lack of training provided for healthcare providers to screen DV cases, thereby weakening their ability to probe DV questions and the lack of empathic listening skills during consultations. As such, DV survivors easily suffer from delayed treatment or referral from physicians [57]. For future endeavors, DV training development can extend the targeted participants to more healthcare professionals who might be poorly equipped, such as new nursing staff, social workers, or counselors during the orientation program.

### HIT Intervention Improved Physical Safety and Psychological Outcomes

HIT interventions have evaluated physical safety and psychological outcomes of women with DV experience. For instance, technology-based interventionshave heightened the awareness of safety strategies among women with DV experience, strengthening their sense of security and capacity to defend themselves from perpetrators within their households [27]. Mobile applications and online training programs have evaluated the current danger level for women, thereby increasing their prompt recognition and engagement in modifying their safety strategies [25]. By leveraging the use of technology, women can actively participate in reassessing their danger level and adapting their contingency plans [27]. They feel safer and find it easier to evaluate their situation on their smartphone [35]. Moreover, online platforms, emails, and messaging systems could reduce the risk of anxiety, depression, and post-traumatic stress disorder (PTSD) among women experiencing DV [25, 39]. Technology provides a valuable platform for women to share and discuss their experiences with other survivors, aligning with previous research indicating that computer-based interventions can significantly improve social support, self-efficacy, and alleviate feelings of loneliness among rural women [58]. Consequently, HIT interventions can contribute to developing a stronger social support network for women facing DV.

There is a wealth of evidence showing the importance of emotional health towards women with DV experiences. However, our review indicated limited HIT intervention studies on assessing the emotional aspects of survivors’ experiences. For example, World Health Organization (WHO) demonstrated that women with IPV experience could have a higher demand for mental health services and an increased likelihood of psychological distress [59]. Women could experience emotional breakdown easier or the need to express themselves after traumatic experiences like DV. The anonymity and instant communication facilitated by mobile devices and the Internet, have provided opportunities for women to seek emotional support through texting through online platforms [60, 61]. This avenue enables better adaptations and enhances the quality of life following traumatic experiences caused by abusive relationships. Future HIT research endeavors could focus on improving the emotional status of survivors, thus promoting more favorable mental health outcomes.

Despite the positive outcomes reported in the literature, adopting HIT interventions for DV population is not without demerits. Several articles have delineated the limitations of using a technical device across community, clinical, and household settings [62, 63]. With reference to the power and control theory of DV, Baddam (2017) believes that the use of technology may exacerbate DV conditions as perpetrators can install tracker applications with GPS and blame the survivors for concealing the DV-related on their smartphones [64]. Perpetrators can turn out to be digital stalkers and constitute a significant threat to the victim, thereby permanently menacing psychological harm to the victim’s life [65].

HIT interventions have predominantly focused on raising awareness and building a digital platform for women to communicate. However, the evaluation of most interventions has typically been limited to pre-and post-test assessments, without consistently tracking long-term performance across multiple time points, such as 3, 6, and 9 months follow-up. Besides, existing research weights physical wellbeing higher than emotional wellbeing. There is currently no standardized measurement for assessing emotions via technology. It is suggested that incorporating advanced machine learning data analytics could be beneficial in extracting emotional needs and delivering emotional support through virtual messaging, such as natural language processing. Further research can capitalize on data analytics to develop an emotional terminology dictionary (e.g., terms related to resilience or distress) that provides a measurable benchmark for extracting and systematically quantifying emotional text data (Table 4).

**TABLE 4:** Literature summary on implications for practice, policy and research among 24 studies From 2008 to 2023 (Worldwide publications, from date of inception to May 2023).

Implications	
For research	➢ Evaluate at different time points ➢ Measure emotion condition ➢ Build ontology and leverage natural language processing to provide intervention ➢ Phase II and III trials (RCT) ➢ Multiple sites RCT
For practice	➢ Web-based interventions in outpatient departments, pre-and-post natal visits, and home visits are more effective than other settings to reach potential women at risk for DV. ➢ Healthcare providers should be trained on DV screening like identifying women’s need in shelter, law protection, child care and family conflict solving. ➢ Technical support should be provided to organizations and clinicians
For policy	➢ Translate the research into clinical and community settings. ➢ More funding is recommended to advance care via technology. ➢ Government should devise a policy to encourage a harmonious, respectful, and amicable environment in our families, workplaces, and communities.

Furthermore, it is important to direct future research avenues towards conducting phase II and III randomized controlled clinical trials to evaluate the preliminary effect size, side effects, and efficacy of the technology within a specific context. The majority of studies reviewed primarily focused on intervention design, feasibility, and prototype intervention. The subsequent step should involve an initial test of intervention in comparison with an appropriate alternative option. To identify outcomes and determine whether the measurement tools can detect the anticipated changes, a small sample size of 40–60 randomized controlled trials should be conducted, enabling the calculation of effect sizes for the intervention [66]. Future research can assess technological interventions by RCT in multiple settings or within a targeted setting to evaluate the efficacy.

This review also provides clinical insights into the context and environment of DV technology interventions. There are abundant web-based interventions, supported by theoretical and empirical evidence of their efficacy and feasibility, to raise awareness, enhance knowledge, and evaluate the specific needs of survivors. The utilization of web-based interventions in outpatient departments, prenatal or postnatal clinics, and home visits yield greater effectiveness compared to other settings. Moreover, existing literature highlights the importance of prioritizing victim’s needs during family conflict. Healthcare providers should be well-informed and trained in addressing the common application procedures and criteria for DV shelter, safety planning, law protection, and childcare through technology (i.e., online training, and case study from online).

The resolution of this public health concern necessitates the implementation of policy modifications or reforms. From a policy standpoint, it is crucial to facilitate the translation of research findings into practical applications in clinical and community settings. In accordance with the socioecological model, the prevention of DV should involve interventions targeting individuals, interpersonal relationships, societal structures, and community dynamics. The government and other relevant stakeholders should join forces and support to violence prevention research endeavors. Additionally, it is recommended to allocate increased funding towards research and innovative technologies to enhance the provision of nursing care and social services through technological interventions. Furthermore, the government should formulate policies to foster an atmosphere of harmony, respect, and amicability within families, workplaces, and communities.

### Limitations

Studies included in this review have methodological considerations regarding the sample size, demographic characteristics, and design that should be noted with caution. Our included studies generally with small sample size, with several studies even being characterized as pilot or exploratory studies. Although the geographical settings from our studies include rural, suburban, urban, clinical, and community areas, the demographic characteristics still predominantly comprise a white population, which affected the overall generalizability of this study. Given the inconsistent reporting of study location among studies, we categorized them as either metropolitan or rural areas based on the current metrics from the U.S. Census Bureau (2016) [67]. Notably, only four studies took place in rural areas; future research should strive to clarify the definition or characteristics of locations where the interventions were conducted. Also, this review did not capture which forms of abuse were experienced by the DV survivors, which could influence the adherence to HIT intervention. We accept this trade-off for this scoping review as the adherence data was not provided in most of the studies. To maintain consistency and precise comparison in the analysis, this review specifically excluded dating violence, and articles using social media to collect data for DV. Therefore, it is possible that certain HIT interventions in DV populations were missed from this review.

### Conclusion

By and large, DV remains a significant public health issue characterized by repetitive trauma within a vicious cycle, especially in the United States. This review highlights the current scope of HIT interventions in addressing DV. Our synthesized literature demonstrates substantial heterogeneity in intervention modalities, target populations, as well as study designs. HIT interventions offer an opportunity to reach potential survivors from underserved rural and suburban areas where access to healthcare services and DV support may be limited. Moreover, the range of HIT interventions for DV encompasses interventions at the patient and provider levels. Implementing online training modules for multidisciplinary healthcare practitioners shows promise in improving outcomes, as demonstrated by pre- and post-intervention assessments. While not all physical health outcomes were measured, the findings emphasize technology’s immense potential in reaching a broader and more heterogeneous sample base. Leveraging HIT can support DV survivors across individuals, families, and communities.
